# Percutaneous Edge-to-Edge Tricuspid Valve Repair in a Patient with Cor Triatriatum Dexter: A Case Report

**DOI:** 10.3390/jcdd8090111

**Published:** 2021-09-14

**Authors:** Fabian Barbieri, Mark Schröder, Niklas Beyhoff, Ulf Landmesser, Markus Reinthaler, Mario Kasner

**Affiliations:** 1 Department of Cardiology, Charité—Universitätsmedizin Berlin, Corporate Member of Freie Universität Berlin, Humboldt-Universität zu Berlin, and Berlin Institute of Health, Campus Benjamin Franklin, 12203 Berlin, Germany; niklas.beyhoff@charite.de (N.B.); Ulf.Landmesser@charite.de (U.L.); markus.reinthaler@charite.de (M.R.); mario.kasner@charite.de (M.K.); 2 Institute of Active Polymers and Berlin-Brandenburg Center for Regenerative Therapies, Helmholtz-Zentrum Hereon, 14513 Teltow, Germany; mark.schroeder@hereon.de; 3 DZHK (German Centre for Cardiovascular Research), Partner Site Berlin, 10785 Berlin, Germany; 4 Berlin Institute of Health (BIH), 10178 Berlin, Germany

**Keywords:** tricuspid regurgitation, cor triatriatum dexter, transcatheter tricuspid valve repair, TriClip, interventional echocardiography, case report

## Abstract

Background: Tricuspid regurgitation is gaining importance due to its high morbidity and mortality. Especially in the elderly, novel technologies in percutaneous therapies have become valuable options due to the commonly present high surgical risk. Case presentation: We report a case of a 78-year-old female suffering from massive tricuspid regurgitation with repetitive right-sided heart failure hospitalizations. As the patient was very frail and deemed as high surgical risk, we used the TriClip^®^ system to improve her symptomatic status. During diagnostic work-up, an additional membrane separating the right atrium, consistent with the definition of a cor triatriatum dexter, was found. Although increasing the complexity of the procedure, implantation of 3 clips with reduction of tricuspid regurgitation to a mild-to-moderate degree was achieved without any notable complications. The patient was discharged with ameliorated symptoms on the fourth postoperative day. Conclusions: Our case highlights the feasibility of percutaneous edge-to-edge tricuspid valve repair in an elderly woman with cor triatriatum dexter. Accurate echocardiographic visualization is an absolute requirement to gain access to the tricuspid valve without interacting with prevailing additional membranes.

## 1. Introduction

Cor triatriatum is a rare congenital malformation defined by subdivision of an atrium into two separate chambers affecting either the right or left side of the heart. In cor triatriatum dexter (CTD), the atrium is typically split into a posterior and an anterior part. The posterior part drains blood of the inferior vena cava (IVC) and includes the fossa ovalis, while the anterior part communicates with the tricuspid valve and forms the estuary of the coronary sinus. The membrane separating both parts of the atrium is widely appreciated as a persistent prominence of the Eustachian and Thebesian valves [1,2]. Partial regression of the right sinus venosus valve with no remaining atrial septal attachments should be differentiated in nomenclature, which can then simply be called the prominent Eustachian valve [3]. In most cases, CTD is not of hemodynamic significance and remains unrecognized during life. Nonetheless, it may be accompanied by right atrial outflow obstruction, e.g., due to tricuspid atresia or pulmonary atresia without ventricular septal defect. In either case, the prominent valves would retain their fetal function by guiding blood flow through the patent foramen ovale, commonly leading to pronounced cyanosis [1,2]. Diagnostic workup of such cyanosis typically results in early recognition of CTD during the neonatal period.

Due to the nature of its morphology, CTD increases the complexity in planning and conducting percutaneous cardiac procedures. We therefore present a case report of TriClip^®^ (Abbott Laboratories, Abbott Park, IL, USA) implantation for massive tricuspid regurgitation (TR) in a patient with an incidentally found CTD.

## 2. Case Description

**Figure 1:**
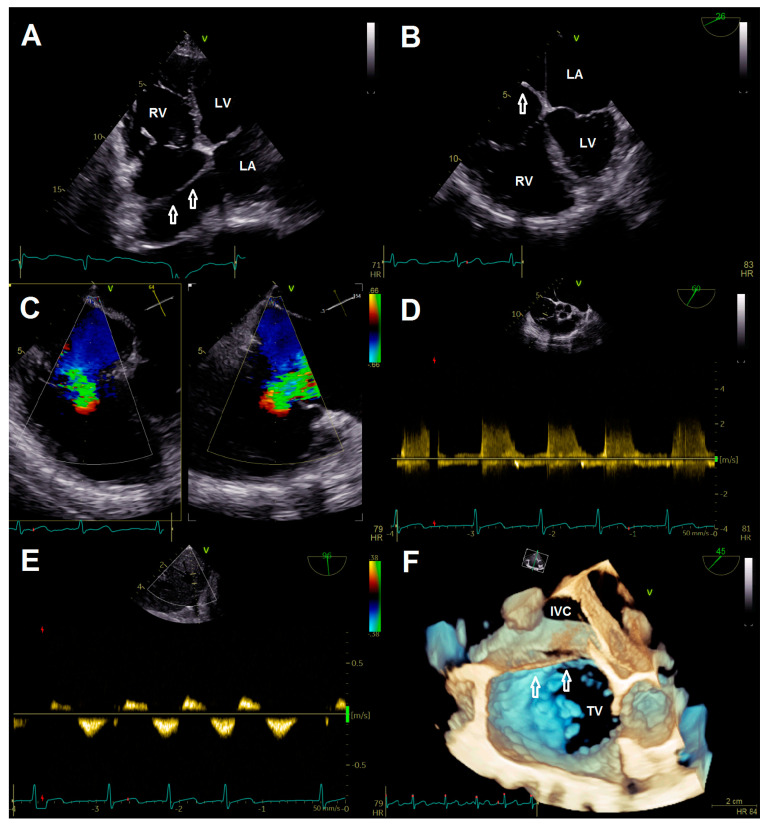
(**A**) Transthoracic four chamber view displaying the additional membrane in the right atrium (arrows). (**B**) Transesophageal four-chamber view demonstrating the membrane (arrow) resulting in a cor triatriatum. (**C**) Multiplane color doppler image revealing massive tricuspid regurgitation. (**D**) Continuous wave doppler showing no significant tricuspid gradient excluding tricuspid stenosis. (**E**) Flow reversal in liver veins; (**F**) 3-D image for visualization of access route. Abbreviations: IVC, inferior vena cava; LA, left atrium; LV, left ventricle; RV, right ventricle; TV, tricuspid valve.

**Figure 2:**
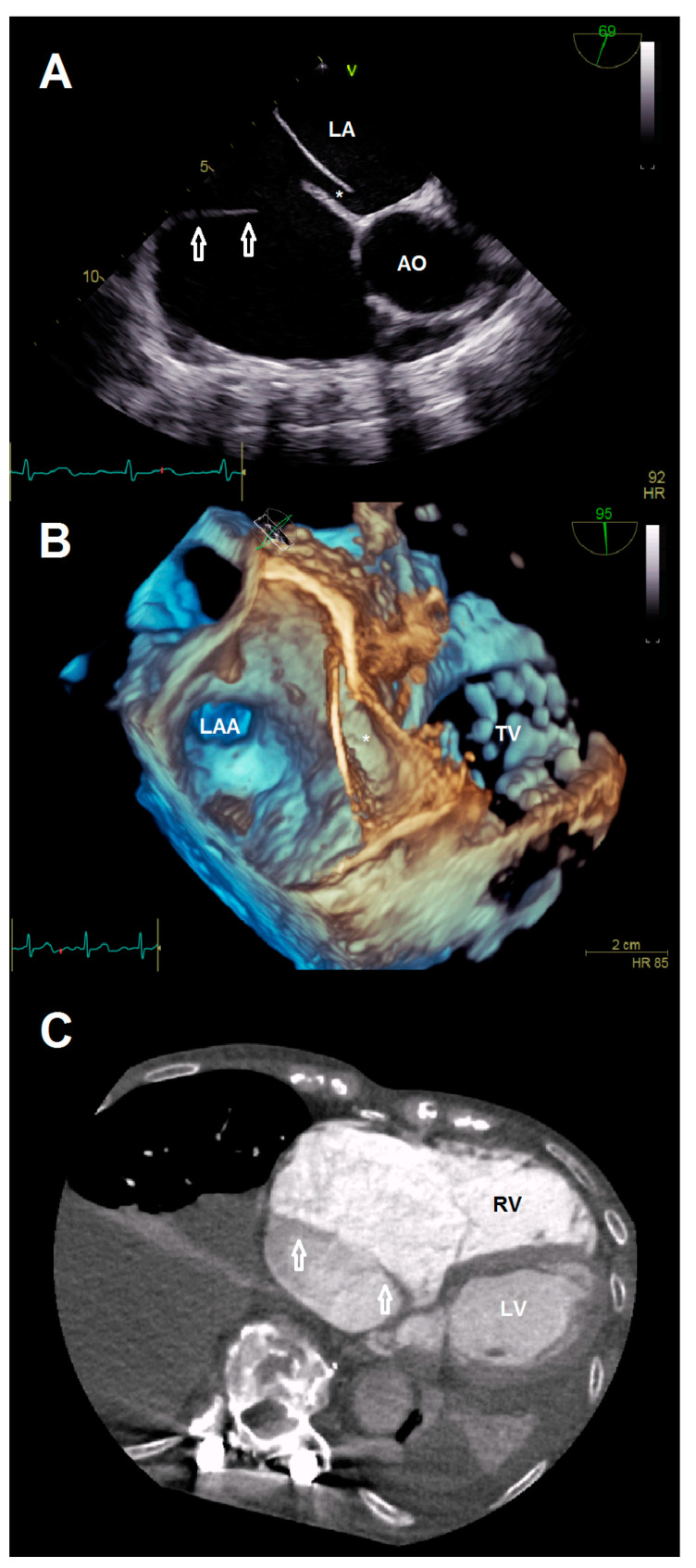
(**A**) Anatomical relationship between the additional membrane (arrows) and present patent foramen ovale (*). (**B**) 3-D view showing the patent foramen ovale (*). (**C**) Cardiac computed tomography visualizing the cor triatriatum dexter (arrows) for procedural planning. Abbreviations: AO, aorta; LA, left atrium; LAA, left atrial appendage; LV, left ventricle; RV, right ventricle; TV, tricuspid valve.

A 78-year-old female patient presented at the emergency department due to progressive dyspnea and a weight gain of 15 kg in recent weeks. Physical examination revealed massive peripheral edema and concomitant congestive dermatitis, but no jugular vein distention. Laboratory results were suggestive for diagnosis of cardiac decompensation with N-terminal pro-brain natriuretic peptide (NT-proBNP) plasma levels of 2320 ng/L, while renal function and inflammation parameters were unremarkable. As the patient was already known to have severe TR with last heart failure hospitalization one year ago, she was admitted for cardiac recompensation and evaluation of tricuspid valve intervention. Further notable medical history included permanent atrial fibrillation, iron deficiency anemia, and severe osteoporosis with previous spondylodesis and extensive oral pain therapy. Echocardiographic examination confirmed progression to massive TR (Vena contracta: 15 mm, effective regurgitation orifice area: 0.9 cm²), but also revealed an additional right atrial membrane ranging from the interatrial septum to the ostium of the IVC consistent with a CTD (Figure 1, Videos S1–S8). Application of contrast medium, as part of the bubble study, yielded an additional patent foramen ovale (PFO, Figure 2A,B; Videos S9 and S10). Besides, both atria (left atrial volume index, 109 mL/m2; right atrial volume index, 189 mL/m^2^), the right ventricle (right ventricular end diastolic diameter, 51 mm), and the IVC (33 mm) were severely dilated, whereas the left ventricular end diastolic diameter (46 mm) was in normal range. Right ventricular (tricuspid annular plane systolic excursion (TAPSE), 23 mm) and left ventricular function (ejection fraction, 60%) were preserved. Furthermore, liver vein congestion with flow reversal and incipient parenchymal liver disease, interpreted as cardiac cirrhosis, were identified underlining the significance of present TR (Figure 1E). Invasive coronary angiography excluded the presence of coronary artery disease, right-heart catheterization determined an isolated postcapillary hypertension (pulmonary artery pressure, 52/17/29 mmHg; wedge pressure, A-wave—20 mmHg, V-wave—23 mmHg, mean—19 mmHg). Cardiac computed tomography was conducted for the purpose of interventional planning (Figure 2C).

As diagnostic work-up was complete, treatment strategies were discussed in the interdisciplinary Heart Team meeting. Due to the frailty of our patient and corresponding high surgical risk, a decision was made opting for a catheter-based repair using the TriClip^®^ system. The patient agreed to intended strategy.

**Figure 3:**
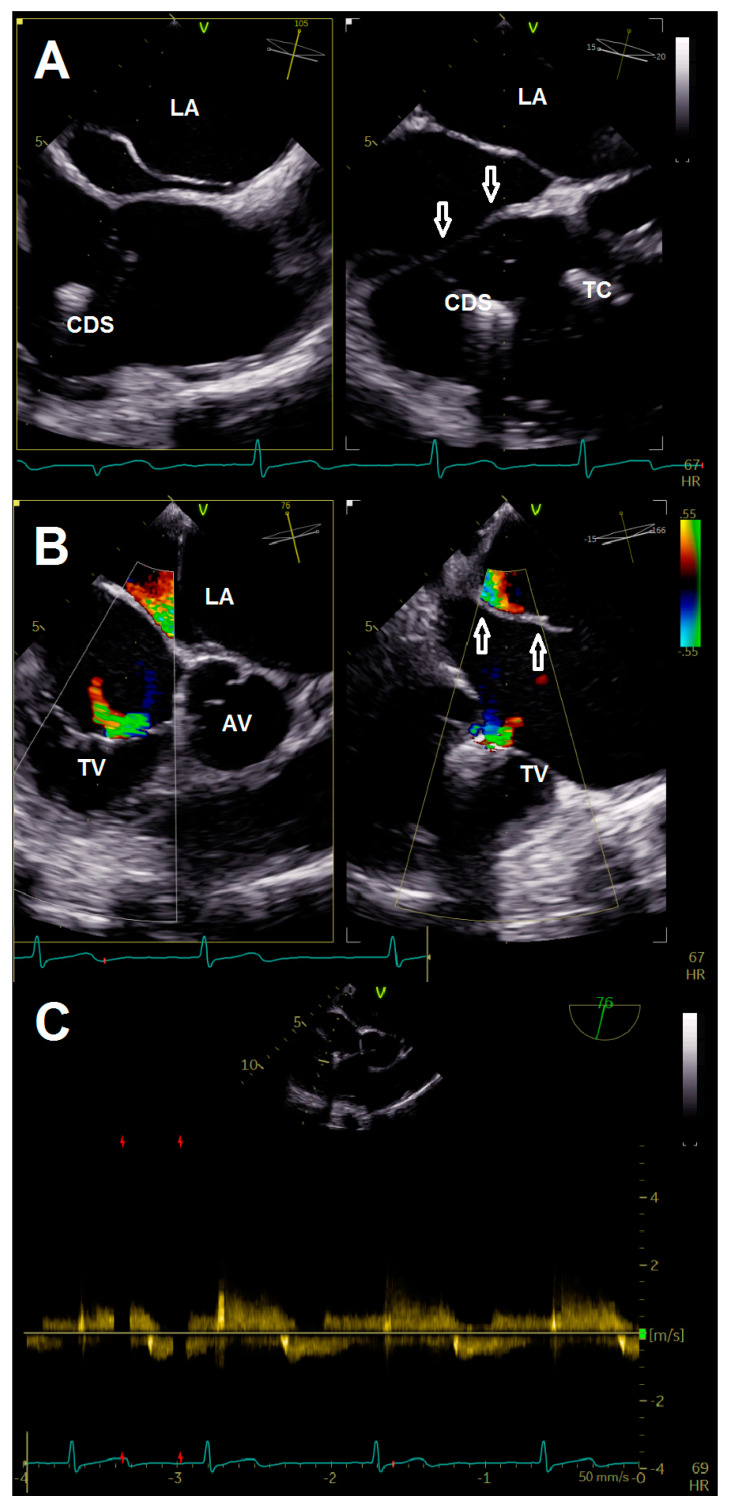
(**A**) Crossing of membrane (arrows) during introduction of the clip delivery system. (**B**) Multiplane color doppler evaluation of tricuspid regurgitation after implantation of three clips. (**C**) Continuous wave doppler after clip implantation showing no relevant tricuspid stenosis. Abbreviations: AV, aortic valve; CDS, clip delivery system; LA, left atrium; TC, TriClip^®^; TV, tricuspid valve.

Intervention was performed with general anesthesia, using the right femoral vein as access route. First, the guiding sheath was positioned in the SVC with careful retraction during the steering maneuver of the clip delivery system (CDS) to avoid interaction with the CTD membranes. After alignment of the CDS and device according to targeted anteroseptal commissure, the first clip (TriClip^®^ G4 XT) was implanted (Videos S11 and S12). Although re-evaluation of TR showed adequate reduction, the remaining jet was still classified as significant (Videos S13–S16). Therefore, we intended to implant two more clips centrally to the first one. Again, great care was taken to maneuver the CDS into the right ventricle by passing the membrane through its orifice (Figure 3A, Video S17). Deployment of both devices (TriClip^®^ G4 XT) was achieved without any notable complications (Videos S18–S26). The anteroseptal commissure was sealed, resulting in bicuspidalization of the tricuspid valve and mild-to-moderate residual TR (Figure 3B, Videos S27–S29). Postprocedural echocardiography confirmed procedural results with a transvalvular mean gradient of 3 mmHg (Figure 3C), while pericardial effusion was excluded. After a few hours of uneventful observation at the intensive care unit, the patient was transferred to the normal ward. She was discharged with improved symptoms on the fourth postoperative day.

Three months later at a short-term follow-up, she described an ongoing improvement in dyspnea. Due to the initial amelioration of symptoms, oral diuretic therapy was reduced by her external physician leading to recurrent leg edema. Corresponding to her fluid retention, we found worsening to severe TR. Accordingly, diuretics were uptitrated again.

## 3. Discussion

TR has become one of the most common valvular heart diseases and increases with age, irrespective of gender. Especially in the elderly, the prevalence of significant TR is estimated in up to 2.7% of patients [4]. Although recent studies have attempted to shed more light on this long-forgotten valve, prognosis remains poor, affecting individuals with common complications such as frequent heart failure hospitalizations, high burden of atrial fibrillation, poor quality of life, and increased mortality [5]. Due to the high prevalence of significant TR with increasing age, many patients are further deemed as high surgical risk, leaving most of them without any other choices than conservative therapy. Thankfully, recent advancements in percutaneous technologies have created novel perspectives, but are yet lacking sufficient data to support their widespread use [6]. One of these emerging devices is the TriClip^®^ system, a leaflet plasty device used for reducing TR by either creating a triple-orifice or a bicuspidalized valve [7]. It is the first-in-class edge-to-edge valve repair system designated for therapy of TR. The main difference to the well-known MitraClip^®^ (Abbott Laboratories) system, which has been used as an off-label device for treatment of TR, is the dedicated guide catheter, which is intended to improve the steering mechanism and therefore facilitate the coaxial approach to the tricuspid valve [8]. The first single-arm trial evaluating its effectiveness showed promising results [9], while a corresponding randomized-controlled trial—the TRILUMINATE pivotal trial (NCT03904147)—is still ongoing. Given the increasing number of implantations worldwide, complex anatomies will be more commonly faced in daily routine. Regarding our case with prevalent CTD, the major difficulty was introducing and maneuvering the CDS into an adequate position for clip implantation without damaging or perforating the separating membrane and its surrounding structures. To minimize this risk, it is recommended to perform the procedure in the presence of accurate echocardiographic visualization by experienced personnel. Preprocedural cardiac computed tomography should be considered to improve anatomical understanding. Furthermore, 3-D printing as a training tool and intraprocedural use of a steerable sheath to test the accessibility of the targeted commissure before introducing the device may be additional options to increase procedural success rates.

Overall, there are few articles describing percutaneous procedures in patients with CTD. Besides three cases, which have demonstrated feasibility of ablation for atrial flutter [10] and atrial fibrillation [11,12], there is also a case series including successful closure of an atrial septal defect, a PFO closure, and a left atrial appendage [13].

## 4. Conclusions

In conclusion, our case highlights the feasibility of percutaneous edge-to-edge tricuspid valve repair in an elderly woman with CTD suffering from recurrent heart failure hospitalizations. Although the presence of CTD enhances the complexity of intervention, the increased risk is mainly limited to gaining access to the tricuspid valve. The procedure seems to be achievable by experienced physicians in the setting of good echocardiographic image quality.

## Data Availability

Data is contained within the article and supplementary material.

## Supplementary Materials

Videos S1–S10: Preoperative evaluation of tricuspid regurgitation, cor triatriatum dexter, and patent foramen ovale. Videos S11–S26: Clip implantation. Videos S27–S29. Final result after implantation of 3 clips.
